# Weyers Acrofacial Dysostosis: A Case Report

**DOI:** 10.7759/cureus.53135

**Published:** 2024-01-29

**Authors:** Aditya M Jain, Amar Taksande, Sarika Gaikwad, Ritwik Nath, Chaitanya Kumar Javvaji

**Affiliations:** 1 Department of Pediatrics, Jawaharlal Nehru Medical College, Datta Meghe Institute of Higher Education and Research, Wardha, IND

**Keywords:** microdontia, curry‑hall syndrome, onychodystrophy, acrodental dysostosis, polydactyly

## Abstract

Weyers acrofacial dysostosis (WAD) is a rare skeletal dysplasia, which is autosomal-dominant, and the clinical symptoms are presented as dental anomalies, polydactyly, nail dystrophy, and short physical stature. It is also termed “Curry‑Hall syndrome” and reported to be linked to genetic mutations mapped on chromosome 4p16, the region reported being commonly associated with a similar genetic syndrome, Ellis-van Creveld (EVC) syndrome. Most individuals with EVC have congenital heart abnormalities, most often atrial septal defects, unlike WAD. In this case, a 15‑year‑old girl presented with onychodystrophy and polydactyly observed in the hands and feet, microdontia, or agenesis of teeth, which were conical in shape, with a short stature. The patient had dystrophy of nails since birth, and physical growth in terms of height did not match the normal growth parameters with respect to age. The patient also had abnormal dentation with conical-shaped teeth, with the rest of the clinical presentations suggestive of WAD.

## Introduction

The dominant hereditary disorder, Weyers acrofacial dysostosis (WAD), also called Curry-Hall syndrome, is characterized by dental and oral malformations, polydactyly of both hands and both feet, nail dystrophy, and small height. It was first reported by Curry and Hall in 1979 [1]. Similarities exist between this ailment and a hereditary disorder termed Ellis-van Creveld (EVC) syndrome, which is also a type of ciliopathy variant [2]. Along with abnormal development of the teeth, bones, and nails, congenital heart problems, short ribs, and thoracic dysplasia have also been observed in individuals with EVC. Both WAD, also known as Curry-Hall syndrome, and EVC syndrome are attributed to heterozygous mutations in the EVC2 and EVC genes situated on chromosome 4p16.2 [3]. EVC2 is recognized to be involved in the cell-to-cell signaling cascade. These pathways are involved in cell growth and differentiation and play an essential part in the regular shaping of the body parts. An abnormal protein EVC2 is produced when there are mutations in genes linked to WAD pathways. Research reports present evidence of EVC2 impeding developmental pathways that lead to abnormal formation and de-shaping of teeth, nails, and bones in growing embryos [4]. WAD is phenotypically like EVC, which is an autosomal recessive disorder but with severe intensity [5,6].

## Case presentation

**Figure 1:**
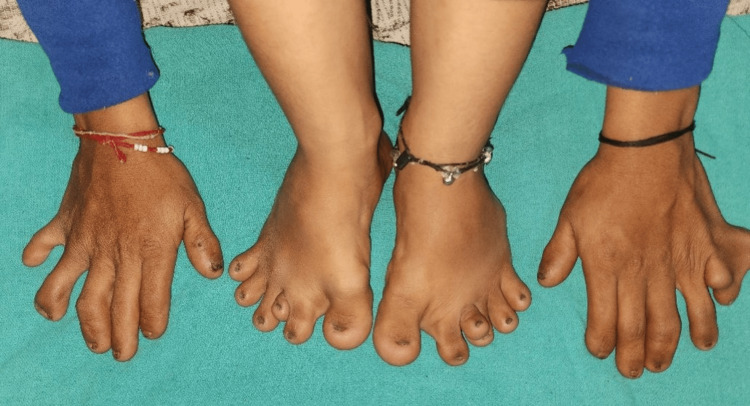
Polydactyly of both hands and both feet with dystrophic hand and toenails

A 15-year-old female visited our hospital with short stature as a major complaint. The child was born to a G5P4L4 mother with a self-reported third-degree consanguineous marriage. The child’s weight and height were observed as 25 kg and 110 cm, respectively, with an expected 55 kg weight and 155 cm height at a z-score <3. The stature of the child was short. It was observed that the child was presented with polydactyly in both hands and both feet, and the fingernails and toenails of the child showed dystrophic changes since birth (Figure 1).

**Figure 2:**
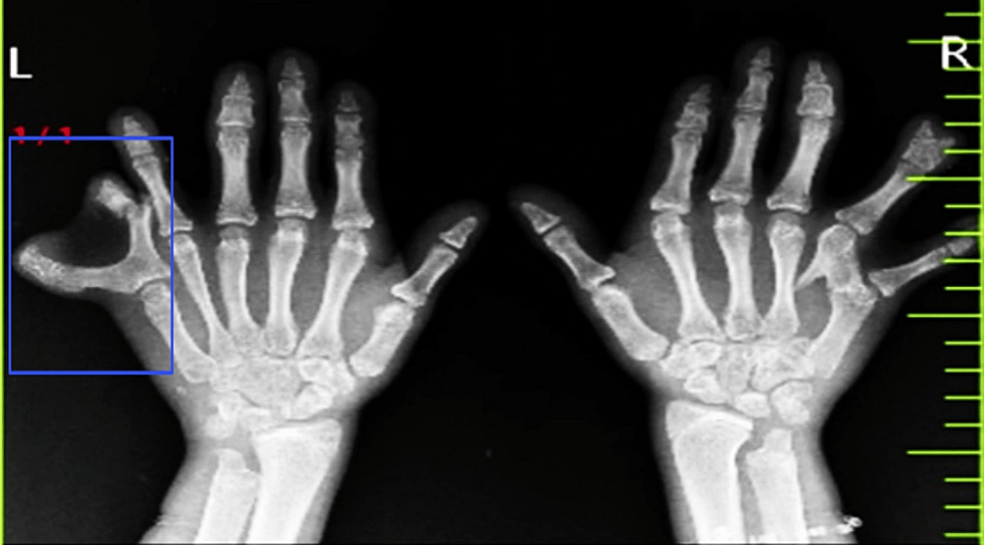
Radiography image of polydactyly in both hands Highlighted area marks polydactyly in the left hand

Figure 2 depicts the X-ray image of the presentation of polydactyly in both hands.

**Figure 3:**
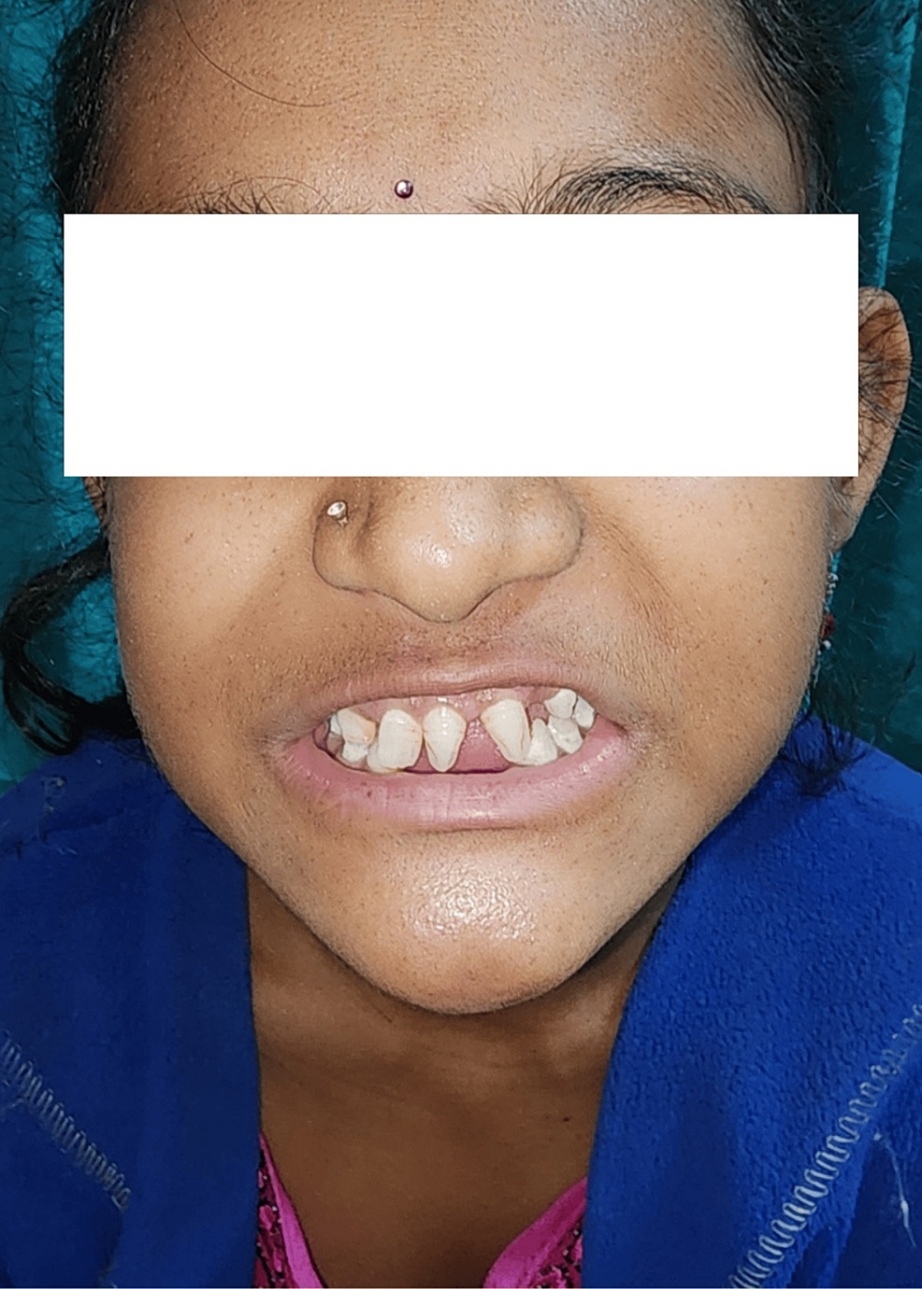
Intraoral view shows abnormal dentation with microdontia with a conical shape

An intraoral examination revealed that the patient had microdontia, and primary incisors were found to be cone-shaped, with primary molars having irregular cusps (Figure 3).

**Figure 4:**
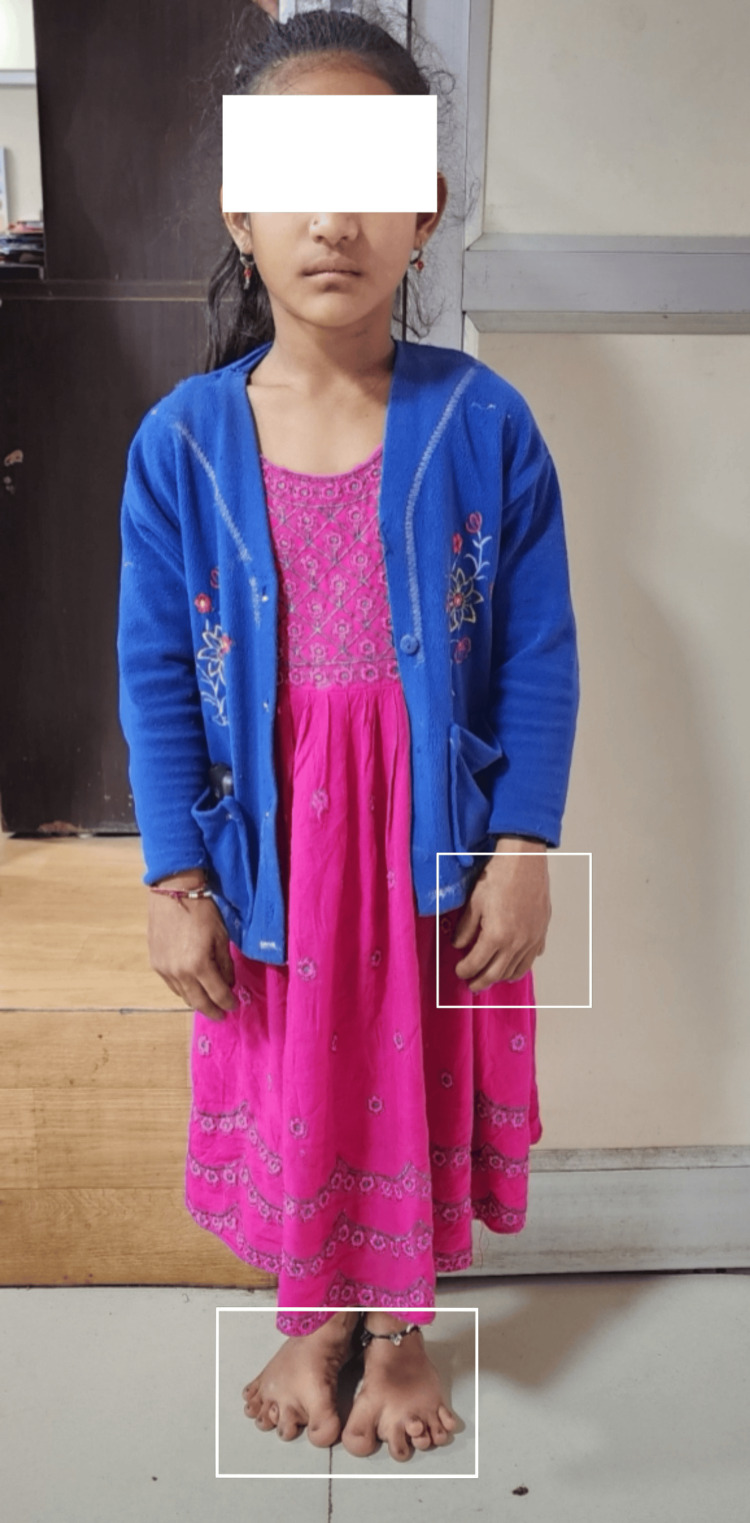
Short stature of the patient with WAD

The child had a short stature with a height that was three standard deviations below the expected height for this age and gender according to the WHO standard growth chart. The upper segment to the lower segment ratio was 0.9 (Figure 4).

The patient had a positive family history of polydactyly (both in hands and in feet) at birth, which was reported by her grandfather. The child was subjected to 2D echocardiography, which reported normal cardiac structure and function. Ultrasonography of the abdo-pelvic region suggested normal functioning, and no renal abnormalities were noted. Physical and clinical presentations were suggestive of WAD. Genetic counseling with regular follow-up was advised.

## Discussion

Other syndromes are reported with mutations in the same genetic loci as WAD. Apart from the clinical presentations mentioned earlier, teeth agenesis or hypodontia and supernumerary teeth have also been mentioned in WAD cases [7]. EVC has similar clinical presentation and mutation in chromosome 4p16.2 is EVC syndrome. A comparative analysis of the clinical presentations of WAD and EVC in this case is presented in Table 1.

**Table 1:** Comparative analysis of Weyers acrofacial dysostosis and Ellis-van Creveld syndrome with current case presentations

Phenotypic features	EVC	WAD	Present case
Microdontia	+	+	+
Conical teeth	+	+	+
Short stature	+	+	+
Polydactyly	Hands sometimes feet	Hands and feet	Hands and feet
Onychodystrophy	+	+	+
Thoracic dysplasia	+	-	-
Cardiac defects	+	-	-
Hair changes	+	-	-
Intelligence	Below normal	Normal	Normal

In multiple studies, linkage and haplotype analysis determined that the disease locus in this pedigree resides on chromosome 4p16, distal to the genetic marker D4S3007 and within a 17-cM region flanking the genetic locus D4S2366 [8,9]. Many of the phenotypic characteristics of EVC and WAD are similar except for its severity. Acrodental dysostosis has been listed in both WAD and EVC except for short stature in WAD in a genetic study by Ruiz-Perez et al. and has been marked as an allelic presentation. The same study reported WAD as a milder presentation than EVC, with most cases having cardiac anomalies [9]. As per a research report on a large subset of Chinese ethnicity, there are novel deletions of autosomal-dominant nature in the EVC2 gene reported to cause WAD, concluding WAD and EVC to be genetically different disorders [8]. In our case, we observed acrodental dysostosis, polydactyly in both the hands and the feet with short stature, and there were no cardiac defects observed, which confirmed the diagnosis of WAD. However, genetic testing confirmation is pending because of the financial constraints faced by the parents. Moreover, short ribs and thoracic dysplasia, which are typical presentations of EVC, were not observed in this case. This patient had polydactyly in both hands and both feet, which is a rare finding in WAD, with only a few cases reported [7].

## Conclusions

In conclusion, the presented case underscores the clinical manifestations and diagnostic challenges associated with WAD, an autosomal-dominant disorder characterized by dental anomalies, nail dystrophy, polydactyly, onychodystrophy, and mild short stature. Despite the potential overlapping features with EVC syndrome, the absence of genotyping and the presence of severe complications such as thoracic dysplasia, cardiac anomalies, and absence of mental retardation favor the diagnosis of WAD. This case highlights the importance of comprehensive clinical and radiological assessments in reaching an accurate diagnosis, shedding light on the distinctive features and potential differentiators between the related syndromes. Further research and genetic studies may contribute to a deeper understanding of the molecular basis and pathophysiology of WAD, facilitating improved diagnostic precision and potentially informing therapeutic interventions for affected individuals.
